# Two Panels of Plasma MicroRNAs as Non-Invasive Biomarkers for Prediction of Recurrence in Resectable NSCLC

**DOI:** 10.1371/journal.pone.0054596

**Published:** 2013-01-16

**Authors:** Céline Sanfiorenzo, Marius I. Ilie, Amine Belaid, Fabrice Barlési, Jérôme Mouroux, Charles-Hugo Marquette, Patrick Brest, Paul Hofman

**Affiliations:** 1 Institute for Research on Cancer and Ageing in Nice IRCAN, INSERM U1081 – CNRS UMR 7284, Team 3, Nice, France; 2 University Hospital Center of Nice, Pasteur Hospital, Department of Pneumology, Nice, France; 3 University of Nice Sophia Antipolis, Faculty of Medicine, Team 3, Nice, France; 4 University Hospital Center of Nice, Pasteur Hospital, Laboratory of Clinical and Experimental Pathology, Nice, France; 5 University Hospital Center of Nice, Pasteur Hospital, Human Biobank, Nice, France; 6 Aix Marseille Université – Assistance Publique Hôpitaux de Marseille, Multidisciplinary Oncology and Therapeutic Innovations Department, Marseille, France; 7 University Hospital Center of Nice, Pasteur Hospital, Department of Thoracic Surgery, Nice, France; University of Barcelona, Spain

## Abstract

The diagnosis of non-small cell lung carcinoma (NSCLC) at an early stage, as well as better prediction of outcome remains clinically challenging due to the lack of specific and robust non-invasive markers. The discovery of microRNAs (miRNAs), particularly those found in the bloodstream, has opened up new perspectives for tumor diagnosis and prognosis. The aim of our study was to determine whether expression profiles of specific miRNAs in plasma could accurately discriminate between NSCLC patients and controls, and whether they are able to predict the prognosis of resectable NSCLC patients. We therefore evaluated a series of seventeen NSCLC-related miRNAs by quantitative real-time (qRT)-PCR in plasma from 52 patients with I-IIIA stages NSCLC, 10 patients with chronic obstructive pulmonary disease (COPD) and 20-age, sex and smoking status-matched healthy individuals. We identified an eleven-plasma miRNA panel that could distinguish NSCLC patients from healthy subjects (AUC = 0.879). A six-plasma miRNA panel was able to discriminate between NSCLC patients and COPD patients (AUC = 0.944). Furthermore, we identified a three-miRNA plasma signature (high miR-155-5p, high miR-223-3p, and low miR-126-3p) that significantly associated with a higher risk for progression in adenocarcinoma patients. In addition, a three-miRNA plasma panel (high miR-20a-5p, low miR-152-3p, and low miR-199a-5p) significantly predicted survival of squamous cell carcinoma patients. In conclusion, we identified two plasma miRNA expression profiles that may be useful for predicting the outcome of patients with resectable NSCLC.

## Introduction

Lung cancer, predominantly non-small cell lung cancer (NSCLC), is the leading cause of cancer-related deaths worldwide [1]. Despite subtle progress over recent years in terms of treatment strategies, the high mortality rate has not decreased significantly. NSCLC is often diagnosed at advanced stages with an overall 5-year survival less than 15% [2]. The poor prognosis of NSCLC patients is largely due to the lack of routine, validated, effective and low cost screening tools that allow detection of early-stage tumors. Developing such biomarkers is a public health imperative since diagnosis and treatment of early-stage NSCLC is associated with 60–80% survival at 5 years [1], [3]. One of the major clinical determinants in NSCLC prognosis is tumor extension, roughly characterized by the pTNM stage. However, a large variability in disease outcome has been observed for a subset of patients with similar clinical and pathological features, thus the current staging system may be insufficient to consistently predict the treatment outcome of NSCLC [4]. Therefore, prognostic assessment of the patients is essential to choose the best therapeutic strategy and may be improved by the integration of new robust prognostic biomarkers.

The impact on outcome of NSCLC of screening procedures such as chest X-rays, sputum cytology, spiral computed-tomography (CT), or a combination of these, has been evaluated in large-scale clinical trials. However, these analyses have not significantly affected overall survival (OS) and have demonstrated low sensitivity [5], [6]. The search for non-invasive tumor biomarkers is rapidly expanding and investigation into circulating biomarkers is the subject of intense research. Several serum tumor markers such as the carcinoembryonic antigen or Cytokeratin-21-Fragment (CYFRA 21-1) may carry some prognostic and predictive information in NSCLC, although their use is currently limited and the biochemical methodologies used to measure them are still labor-intensive [7], [8].

One of the most exciting molecular markers in tumor diagnosis and prognosis are microRNAs (miRNAs) [9]. miRNAs are small RNA molecules (18 to 24 nt) that effect substantially the expression of multiple genes at a post-transcriptional level, via mRNA destabilization or translational repression [10]. Deregulation of miRNA expression is thought to be responsible of tumor initiation and progression [11]. MiRNAs are frequently deregulated in cancer and may act as oncogenes or tumor suppressors having regulatory functions on hundreds of downstream genes with different biologic functions [12], [13]. Given the fundamental role of miRNAs in tumors and their global deregulation, miRNA profiles may provide a more accurate prediction of survival than the expression of a single-marker or expression profiles of protein-coding genes [13]. In addition, recent studies have demonstrated that specific expression profiles of circulating miRNAs could be promising blood-based non-invasive biomarkers useful for cancer detection and prognosis in different types of cancer, including NSCLC [14], [15], [16], [17], [18]. Human serum or plasma contains a large amount of intact and stable miRNAs, which can be detected with a simple assay such as quantitative real-time PCR (qRT-PCR) [19]. Therefore, the high stability of miRNAs allows for efficient identification in various clinical specimens including sputum, plasma, serum, and frozen and formalin-fixed paraffin embedded tissue samples [14], [20], [21], [22].

The aim of our study was to: 1) select a large panel of miRNAs that have been reported to be highly deregulated in NSCLC, 2) determine whether the plasma expression profiles of these miRNAs were altered in NSCLC patients compared to healthy individuals and 3) evaluate whether the miRNA profile is able to predict the prognosis of resectable NSCLC. We evaluated a panel of seventeen miRNAs by qRT-PCR in the plasma of 52 patients with resectable NSCLC and 30 controls, 10 patients with chronic obstructive pulmonary disease (COPD) and 20-age, sex and smoking status-matched healthy individuals.

## Materials and Methods

### Study population

Sixty-two patients hospitalized from March 2008 to March 2010 at the Pasteur Hospital (Departments of Pulmonary Medicine, and Thoracic Surgery, CHU de Nice, France) were enrolled in this study. Among these patients, 52 patients had NSCLC and 10 had COPD. COPD patients did not have symptoms of lung cancer or other malignancies. The diagnosis of NSCLC patients was based on examination of all tumor specimens using the 7^th^ pTNM classification and on the last histological classification of NSCLC [23]. In addition, twenty-age, -sex and -smoking status-matched healthy volunteers participated in this study. Written informed consent was obtained from participants after explaining the nature of the study, which was approved by the research ethics board of the Nice University hospital and was performed according to the guidelines of the Declaration of Helsinki. The main clinical and pathological data are summarized in Table 1. Enrollment of patients in our study was conditioned by stringent criteria such as obtained signed consent, availability of resected surgical specimens along with plasma samples, good quality RNA and minimum 18 months follow-up for surviving patients.

**Table 1:** Clinicopathological characteristics of the 52 NSCLC patients, 10 COPD patients and 20 healthy individuals included in our study.

Variables	NSCLC patients n (%)	COPD patients n (%)	Healthy subjects n (%)
**Overall**	52 (100%)	10 (100%)	20 (100%)
**Age** (***years***)			
**Mean ± SD**	65.1±11.1	68.9±6.7	67.5±5.3
**Sex**			
**Male**	39 (75%)	8 (80%)	14 (70%)
**Female**	13 (25%)	2 (20%)	6 (30%)
**Smoking status**			
**Never smoked**	8 (15%)	2 (20%)	5 (25%)
**Former or current smokers**	44 (85%)	8 (80%)	15 (75%)
**Histological type**			
**Adenocarcinoma**	27 (52%)	n/a	n/a
**Squamous cell carcinoma**	25 (48%)	n/a	n/a
**pTNM stage**			
**IA**	8 (19%)	n/a	n/a
**IB**	14 (33%)	n/a	n/a
**IIA**	5 (12%)	n/a	n/a
**IIB**	8 (19%)	n/a	n/a
**IIIA**	7 (17%)	n/a	n/a
**Histologic grade**			
**Well**	22 (42%)	n/a	n/a
**Moderate**	19 (37%)	n/a	n/a
**Poor**	11 (21%)	n/a	n/a
**Adjuvant treatment**	21 (40%)	n/a	n/a
**Life status**			
**Alive**	40 (77%)	10 (100%)	20 (100%)
**Deceased; lung cancer**	9 (17%)	n/a	n/a
**Deceased; other cause**	3 (6%)	n/a	n/a

### miRNA isolation

Peripheral blood (5 ml) was taken prior to surgery and kept in an EDTA-containing tube. The samples were centrifuged at 3000 rpm at 4°C for 10 minutes within 4 hours of collection. The plasma was collected and stored at −80°C until use. Total RNA containing small RNA was extracted from 100 µl of plasma using the miRNeasy Mini Kit (Qiagen GmbH, Hilden, Germany) according to the manufacturer's protocol. The concentration and purity of the RNA were determined with a NanoDrop 1000 (Thermo Fisher Scientific, Wilmington, DE).

### Selection of control genes for quantification of plasma miRNAs

To select good candidates, we used some guidelines from Exiqon company (http://www.exiqon.com/ls/Documents/Scientific/microRNA-serum-plasma-guidelines.pdf) Endogenous controls such as U6, RNU19, miR-16-5p, miR-192-5p, and miR-103a-3p were analyzed in these samples to identify a small RNA expressed at a similar level in equal volume of sera from both healthy subjects and patients with cancer to serve as a normalization control. Only miR-16-5p, miR-192-5p, and miR-103a-3p were expressed at a high level in the samples of this study (median Ct<30; 100%, 100% and 85% detection, respectively) and not statistically different between the analyzed classes (*t*-test; *P*>0.05), and their levels were the least variable for the miRNAs in all samples (SD<0.9) (data not shown). Moreover, miR-103a-3p and miR-16-5p were used as markers of hemolysis [24].

### Analysis of the miRNA expression level

Normalization of the results between patients was performed by substracting the mean of miR-192-5p and miR-16-5p levels to all data (ΔCT) as previously described for other cancers [25], [26], [27]. Thus, the global mean of the relative expression of each miRNA was calculated and subtracted in order to have all miRNA centered on zero for further studies (ΔΔCT). For diagnosis-related analysis, the mean was based on the cohort of healthy controls ΔCT. For the prognosis-related study, the control ΔCT values were removed for the analysis.

### Statistical Analysis

The statistical analyses were performed with SPSS 16.0 statistical software (SPSS Inc., Chicago, IL). Hierarchical clustering and pictures were generated using MeV (TM4 Microarray Software) [28]. The receiver–operator characteristic (ROC) curve and AUC analyses were used to determine the accuracy of each miRNA profile in a specimen with a given specificity rate and to determine the optimal cut-off point. We categorized each miRNA as high or low using the median value as the cut-off. The *chi2*, Student or Mann-Whitney *U*-test tests were used to analyze the correlation between the miRNA expression levels and clinicopathological features of the patients. To assess the association of miRNA expression with disease-free survival (DFS), the Kaplan-Meier method and the log-rank test were used to compare survival times between groups. A Cox proportional hazards model was created to identify independent predictors of survival. Variables that were associated with survival with a *P*-value<0.20 in the univariate analysis were included in the multivariate Cox regression. All *P*-values shown were two sided, and a *P*-value≤0.05 was considered statistically significant.

## Results

### Levels of expression of plasma miRNAs

Based on the literature, we selected seventeen miRNAs reported to be most frequently altered in primary NSCLC patients (Table S1). To determine whether aberrations in the specified miRNAs could be confirmed in independent plasma samples, we assessed expression of the candidate miRNAs in duplicate assays by qRT-PCR for 52 NSCLC plasma samples and 30 control samples (20 healthy subjects and 10 patients with COPD).

Thirteen (76%) miRNAs including the overexpression of miR-20a-5p, miR-25-3p, miR-155-5p, miR-191-5p, mir-223-3p, miR-296-5p, and miR-320-3p along with the underexpression of let-7f-5p, miR-24-3p, miR-126-3p, miR-145-5p, miR-152-3p, miR-199a-5p, were consistently observed in all plasma samples (ΔΔCT<32) (Table 2). No expression was detected for miR-96-5p, miR-129-5p, miR-373-5p, and miR-516-5p, and these miRNAs were excluded from further analyses (Table 2).

**Table 2:** Plasma expression levels of candidate miRNAs in all sample sets.

miRNA	CT Mean	SD	Deregulation in NSCLC cases
**Let-7f-5p**	30.76	2.99	Down-regulated
**miR-20a-5p**	23.08	2.39	Up-regulated
**miR-24-3p**	23.93	2.97	Down-regulated
**miR-25-3p**	24.29	1.80	Up-regulated
**miR-126-3p**	24.20	2.46	Down-regulated
**miR-145-5p**	31.18	3.01	Down-regulated
**miR-152-3p**	28.41	2.75	Down-regulated
**miR-155-5p**	30.50	1.80	Up-regulated
**miR-191-5p**	25.21	2.88	Up-regulated
**miR-199a-5p**	30.25	3.61	Down-regulated
**mir-223-3p**	22.54	3.21	Up-regulated
**miR-296-5p**	32.16	2.09	Up-regulated
**miR-320-3p**	22.62	2.18	Up-regulated
**mir-96-5p**	undetectable	undetectable	undetectable
**mir-129-5p**	undetectable	undetectable	undetectable
**mir-373-5p**	undetectable	undetectable	undetectable
**mir-516-5p**	undetectable	undetectable	undetectable

### MiRNA profiles of NSCLC patients and cancer-free controls

The hierarchical clustering based *t*-test along with the ROC curves were constructed to estimate the sensitivity and specificity of the 13-plasma miRNA panel. The accuracy was 79.4% with a sensitivity of 79% and a specificity of 71% (data not shown).

**Figure 1:**
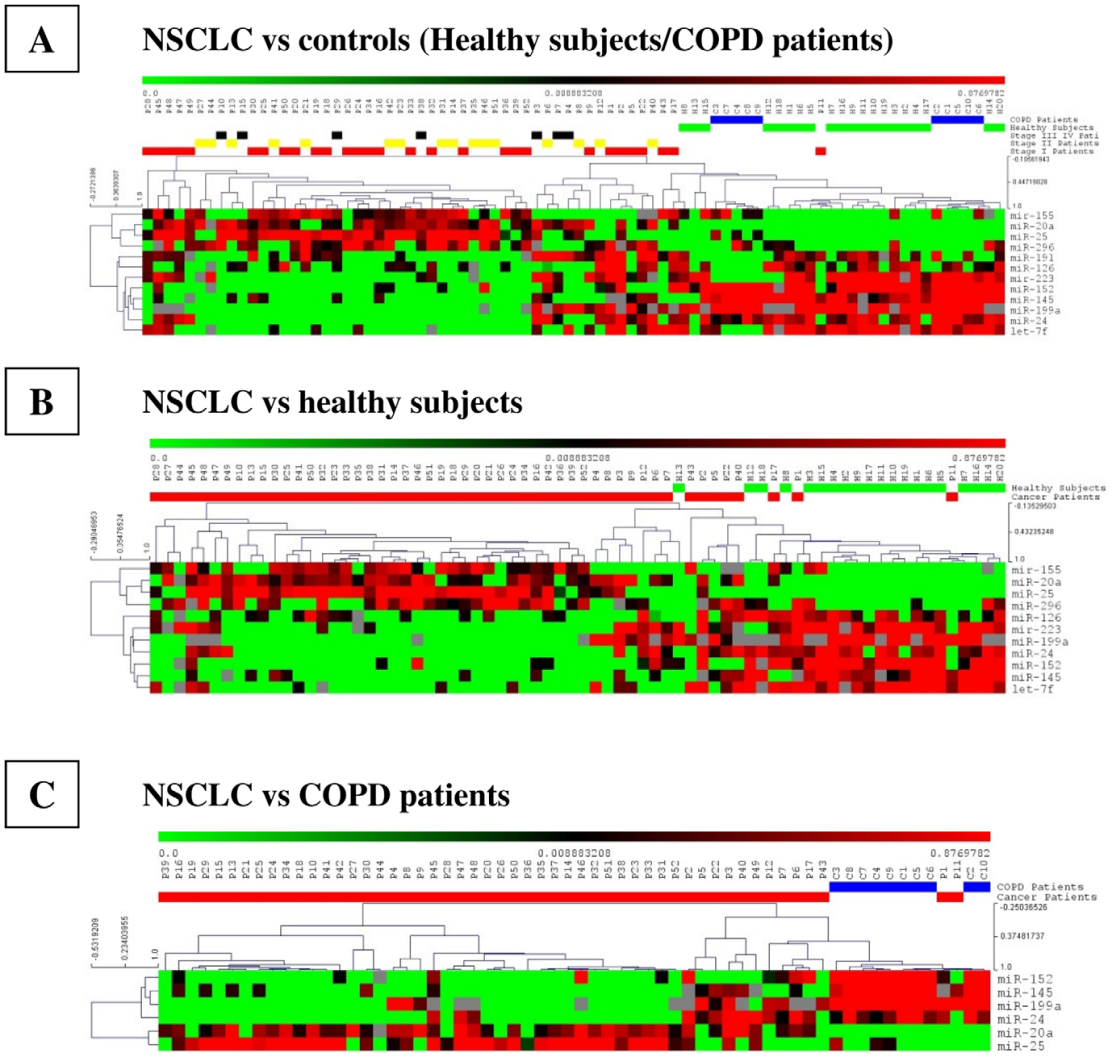
Heat-map clustering analysis of the deregulated miRNA expression levels of NSCLC patients, COPD patients and healthy individuals. Average linkage and 1-Pearson correlation as distance metric were used for the clustering.

However, the ANOVA test, along with the ROC curve estimation yielded a 12-miRNA signature with improved accuracy in discriminating between cancer-free controls and NSCLC patients (Figure 1). The expression of the twelve-plasma miRNA panel including miR-155-5p, miR-20a-5p, miR-25-3p, miR-296-5p, miR-191-5p, miR-126-3p, miR-223-3p, miR-152-3p, miR-145-5p, miR-199a-5p, miR-24-3p, and let-7f-5p allowed significant discrimination between controls and NSCLC patients with an accuracy of 82.1% (95% CI: 0.792–0.850; *P*<0.001), demonstrating a sensitivity of 85% and a specificity of 75% (Figure 1). The twelve plasma miRNAs significantly discriminated between controls and stage I NSCLC patients (AUC = 0.806; Figure 1), stage II NSCLC cases (AUC = 0.849; Figure 1) and stage III NSCLC patients (AUC = 0.878; Figure 1).

Next, we carried out pairwise group comparisons to identify miRNAs that contribute significantly to the different separations, including NSCLC versus healthy controls, NSCLC versus COPD patients, and lung adenocarcinoma or squamous cell carcinoma patients versus either healthy individuals or COPD patients. There was a clear separation of the NSCLC patients from the healthy subjects based on an 11-plasma miRNA profile with an accuracy, sensitivity, and specificity of 87.9%, 81.1%, and 82.9%, respectively (Figure 1). In addition, the diagnostic sensitivity of the 11-plasma miRNA signature was higher for squamous cell carcinoma (91.3%) than for adenocarcinoma cases (85.7%) (*P*<0.05; Figure S1).

Furthermore, a small subset of only six miRNAs still separated NSCLC from COPD with an accuracy, sensitivity, and specificity of 94.4%, 90.9%, and 83.3%, respectively (Figure 1). Interestingly, adenocarcinoma and squamous cell carcinoma cases shared five miRNAs when compared to COPD patients, including miR-20a-5p, miR-152-3p, miR-145-5p, miR-199a-5p, and miR-24-3p. However, miR-191-5p identified only adenocarcinoma patients versus COPD patients, and miR-25-3p, squamous cell carcinoma cases only (Figure S1 & S2). Finally, only three plasma miRNAs were differentially expressed when comparing adenocarcinoma and squamous cell carcinoma patients. Higher plasma levels of miR-20a-5p (*P* = 0.034) and miR-25-3p (*P* = 0.013) along with lower levels of miR-191-5p (*P* = 0.008) were observed in squamous cell carcinoma (Figure S2).

### Correlation between plasma miRNAs and clinicopathological features of NSCLC

We then compared the plasma levels of miRNAs with patient clinicopathological parameters. Higher plasma levels of miR-20a-5p (*P* = 0.012) and miR-25-3p (*P* = 0.04) along with decreased levels of miR-191-5p (*P* = 0.023) were observed in squamous cell carcinoma (Table S2; Figure S2). No significant association was found between the levels of miRNAs and age, sex, history of smoking, tumor grade, and pathological stage (*P*>0.05, Table S2).

### Association of plasma miRNAs with DFS of NSCLC patients

We further investigated whether the expression of plasma miRNAs correlated with DFS in our group of NSCLC patients. The mean DFS in our study population was 46 months (95% CI, 39.4 to 52.9). In the univariate analysis, the clinical factor that significantly associated with DFS was the pTNM stage (*P*<0.0001). The unadjusted survival analysis showed that high plasma levels of miR-155-5p (*P* = 0.068) and miR-20a-5p (*P* = 0.018) along with a low level of miR-152-3p (*P* = 0.049) were associated with poor DFS of NSCLC patients (Figure S3). The remaining miRNAs such as miR-223-3p (*P* = 0.348), miR-191-5p (*P* = 0.671), miR-320-3p (*P* = 0.322), miR-126-3p (*P* = 0.131), miR-145-5p (*P* = 0.705), miR-199a-5p (*P* = 0.612), miR-24-3p (*P* = 0.364), miR-25-3p (*P* = 0.816), miR-296-5p (*P* = 0.853), and let-7f-5p (*P* = 0.964) were not associated with survival (Figure S4).

**Figure 2:**
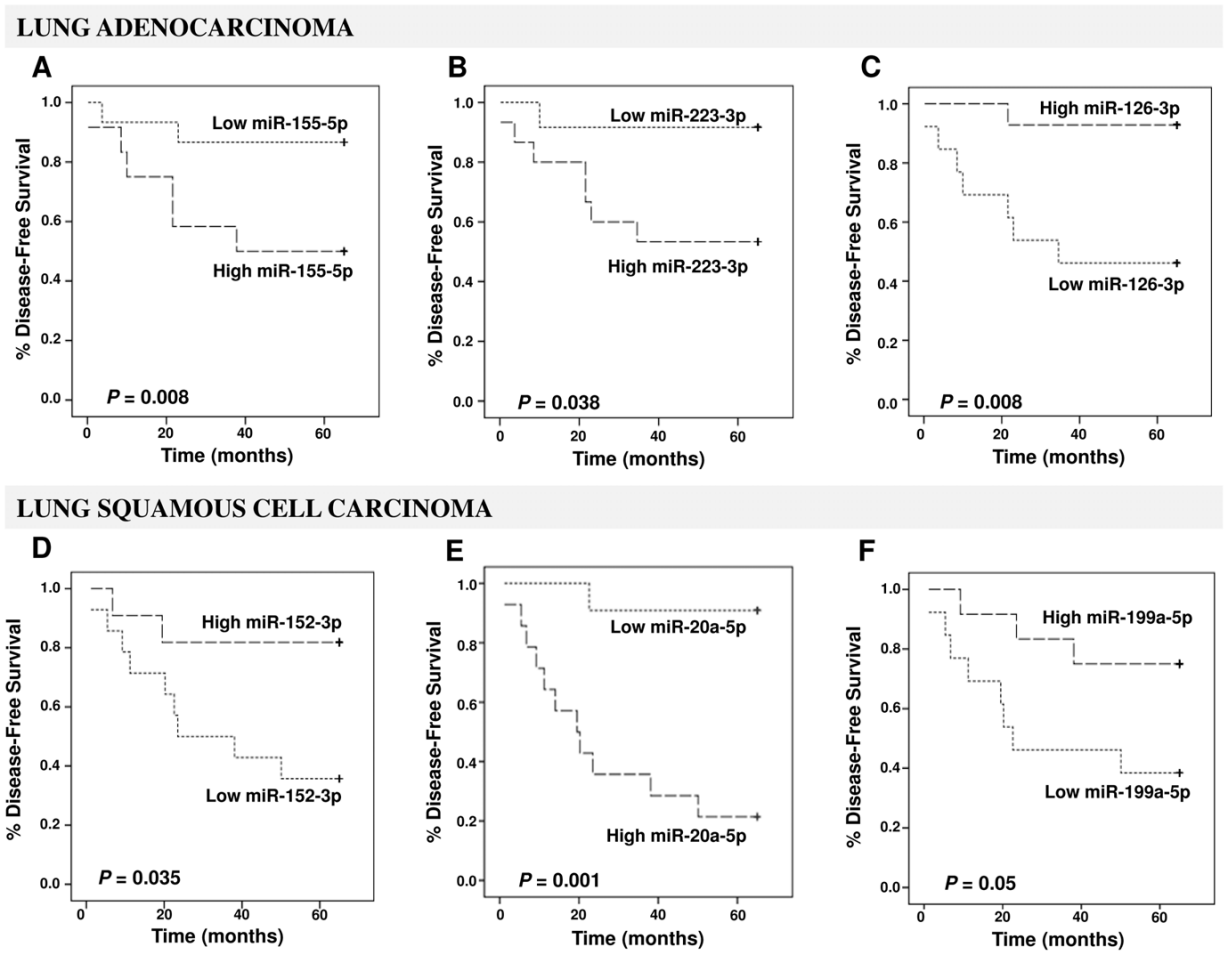
Kaplan-Meier DFS curves for lung adenocarcinoma patients (***upper panels***) **and lung squamous cell carcinoma patients** (***lower panels***) **stratified according to plasma levels of miR-155-5p** (**A**)**, miR-223-3p** (**B**)**, miR-126-3p** (**C**)**, miR-152-3p** (**D**)**, miR-20a-5p** (**E**)**, and miR-199a-5p** (**F**)**.** The *P*-values were calculated using the log-rank test between patients with high- and low-fold changes.

Due to the biological differences in the miRNA expression the survival analyses were conducted separately for adenocarcinoma and squamous cell carcinoma patients. Interestingly, the high plasma levels of miR-155-5p (*P* = 0.008), and miR-223-3p (*P* = 0.038) with low plasma level of miR-126-3p (*P* = 0.008; Figure 2) were significantly associated with poor DFS in lung adenocarcinoma patients. In addition, low plasma levels of miR-152-3p (*P* = 0.035) and miR-199a-5p (*P* = 0.05) along with a high plasma level of miR-20a-5p (*P* = 0.001; Figure 2) significantly correlated to decreased DFS in lung squamous cell carcinoma patients. Age, gender, history of smoking, histological subtype, and tumor grade were not associated with DFS. In a multivariate analysis, the independent factors for improved DFS were stage I (*P*<0.001), low plasma levels of miR-155-5p (*P* = 0.030) and miR-20a-5p (*P* = 0.048) along with high plasma levels of miR-152-3p (*P* = 0.029) and miR-199a-5p (*P* = 0.038) (Table 3).

**Table 3:** Multivariate analysis of prognostic factors identified in our study with DFS as the end point in patients with NSCLC.

Prognostic factor	HR^1^	95% CI^2^	*P*-value^3^
**pTNM stage**			
I vs. II+III	0.095	0.030–0.303	<0.001
**miR-155-5p**			
Low vs. high	0.060	0.005–0.767	0.030
**miR-223-3p**			
Low vs. high	1.449	0.581–3.614	0.426
**miR-20a-5p**			
Low vs. high	2.881	1.009–8.227	0.048
**miR-152-3p**			
High vs. low	0.333	0.125–0.892	0.029
**miR-126-3p**			
High vs. low	0.497	0.191–1.295	0.153
**miR-199a-5p**			
High vs. low	0.204	0.045–0.918	0.038

1 HR; hazard ratio.–^2^CI; confidence interval. ^3^ *P*-value<0.05 statistically significant.

## Discussion

Despite recent advances in diagnosis and treatment strategies, the prognosis of NSCLC across all stages remains unchanged and early detection and prediction of outcome is critical in improving survival. However, it can be sometimes difficult to obtain tissue for diagnosis, in particular in patients with metastatic lung cancer.

Profiling of miRNA expression in lung tumor tissues discriminated cancer patients from cancer-free individuals, and specific miRNAs correlated with disease diagnosis and clinical outcome [29]. Therefore, developing minimally invasive methods by integrating the recent advances in the field of miRNAs for early diagnosis and prognosis of NSCLC is of great interest. Accumulating reports suggest that unique patterns of circulating miRNAs may act as novel biomarkers for early detection of lung cancer and for prediction of outcome [4], [19], [30]. Endogenous circulating miRNAs are stable and resistant to RNases [14], [21]. Because of the simplicity and reproducibility of getting a blood sample, the levels of easily testable miRNAs in plasma seem suited to surveillance of NSCLC outcome [4]. However, it seems that the expression of a single miRNA may not be a reliable biomarker for cancer diagnosis and prognosis [31], [32]. Simultaneous assessment of a panel of tumor-specific circulating miRNAs may improve the sensitivity and specificity for diagnosis of lung cancer and may better predict development of the cancer. Therefore, the investigation of a plasma miRNA signature in NSCLC patients using a qRT-PCR assay, as shown in this study, may be of great clinical interest as a routine procedure.

In our study, an 11-plasma miRNAs signature significantly discriminated healthy individuals from NSCLC patients. The accuracy, sensitivity, and specificity for NSCLC detection by the 11-plasma miRNA panel are 87.9%, 85% and 82.9%, respectively, which are higher to those of blood-based single biomarker, such as CYFRA 21-1 (AUC≈0.84, sensitivity≈50%, specificity≈95%), tissue polypeptide specific antigen (AUC≈0.74, sensitivity≈34%, specificity≈95%), and CEA (AUC≈0.8, sensitivity≈53%, specificity≈95%) [33]. Interestingly, the diagnostic sensitivity of the 11-plasma miRNA signature was higher for squamous cell carcinoma than cases of adenocarcinoma.

It has been widely demonstrated across studies that the miRNA expression profiles strongly differentiate lung adenocarcinoma from squamous cell carcinoma [16]. However, a great number of miRNAs are shared in both histological types of NSCLC, as previously reported, which seems to be the case for our selected miRNA panel [16], [34], [35]. In addition, only three plasma miRNAs were differentially expressed when comparing adenocarcinoma and squamous cell carcinoma patients. In our study, higher plasma levels of miR-20a-5p and miR-25-3p along with lower levels of miR-191-5p were observed in squamous cell carcinoma, as previously reported [16], [35], [36], [37]. Therefore, although most miRNA expression differences were similar for both tumor types, our limited panel of miRNAs still showed fine differences that suggested that the NSCLC subtypes may follow subtle different pathways to tumorigenesis, as previously suggested [38].

Moreover, miRNA detection in plasma may be an effective procedure for the early detection of NSCLC in high-risk patients with COPD. COPD, along with tobacco smoking, is not only a common lung cancer co-morbidity but it is also associated with a higher risk of development of lung cancer [39], [40]. In our study we found six miRNAs differentially expressed in plasma of NSCLC patients when compared to COPD patients, which is consistent with previous reports [39], [41]. This finding could represent a powerful clinical application of the six-miRNA molecular classifier in COPD patients, which has been explored as a method for early diagnosis of questionable lung densities [39], [41].

Although comparison of smokers and never smokers did not demonstrate significant results in our study, possibly because of the small number of never smokers, it was interesting to note that three of the miRNA of the six-plasma miRNA panel, including miR-152-3p, miR-199a-5p and miR-20a-5p, differentially expressed between COPD patients and squamous cell carcinoma patients were able to significantly predict cancer relapse, as previously reported [16], [35], [36], [37]. Our findings emphasize the potential role of these miRNAs as plasma biomarkers playing an important role in lung tumorigenesis and squamous cell carcinoma progression. Finally, we identified a three-plasma miRNA signature including high plasma levels of miR-155-5p and miR-223-3p with low plasma level of miR-126-3p associated with a higher risk for lung adenocarcinoma progression.

Several recent studies have proposed sets of plasma miRNAs as potential markers to monitor the development of lung cancer and its prognosis, in particular for NSCLC [4], [14], [17], [19], [30]. However the results of different studies are quit variable and often identified different miRNAs signatures. The reason for this variability is complex, but likely arises from the differences in patients' ethnicities, sample subtypes (plasma vs. serum vs. whole blood), sample collection methods, technology platforms (microarray or qRT-PCR), as well as the bioinformatic analyses, as previously suggested [42]. We therefore carefully designed our study in order to ensure the identification of reliable miRNAs as plasma biomarkers and employed established methods that reveal valuable clinical information. We only analyzed Caucasian patients to reduce the effect of ethnicity. NSCLC patients were matched according to histology in order to minimize pathological subtype effects and to amplify the molecular homogeneity of tumor specimens. The patients were strictly selected from individuals who had not been previously treated with neoadjuvant treatment to avoid therapy bias. In addition, we demonstrated that our markers could be conveniently measured by qRT-PCR in plasma.

Interestingly, the two panels of plasma miRNAs comprising our lung squamous cell carcinoma and adenocarcinoma progression classifiers have been reported individually to have a prognostic impact in other studies.

Several studies have recently validated the prognostic utility of the high plasma levels of miR-20a-5p in lung squamous cell carcinoma patients [16], [35], [36], [37]. Furthermore, in our study, miR-199a-5p was a consistent element of the plasma miRNA signature for NSCLC diagnosis in comparison to both healthy individuals and COPD patients. This finding was in agreement with previous results obtained in a tobacco-specific carcinogen-induced lung cancer model that suggested a key role of miR-199a-5p as a part of an early-response miRNAs signature associated with pulmonary tumorigenesis [43]. Chen *et* *al*. reported a profiling study on serum miRNA expression among 400 NSCLC cases and 220 controls. MiR-152-3p and miR-223-3p were among the 10-serum miRNAs differentially expressed between cancer patients and control subjects in their cohort [44]. In addition, the serum expression level of miR-223 was associated with cancer-specific mortality in stage IA/B NSCLC patients [17]. Moreover, by negatively regulating tumor-suppressor genes, miR-155-5p promotes malignant transformation and cancer progression in many types of cancer, including NSCLC [45], [46]. In our study, the up-regulation of miR-155-5p was found to be an independent negative prognostic factor in lung adenocarcinoma patients, as previously reported [34], [47]. Shen *et* *al*. reported a four-miRNA panel in plasma, including miR-126 that distinguished NSCLC from healthy controls with a sensitivity and specificity of 73% and 96%, respectively [30]. One study reported the role as prognostic biomarker for miR-126-3p in NSCLC patients [48]. Finally, the fact that the six plasma miRNAs identified with prognostic utility in our study are common to other studies of NSCLC patients validates our findings.

In conclusion, we developed a robust methodology to study miRNAs in the plasma of NSCLC patients by using a clinically applicable qRT-PCR method. We identified two-plasma miRNA signatures-histology related that are highly predictive of cancer progression. Three-plasma miRNA panel, including miR-152-3p, miR-199a-5p and miR-20a-5p, was associated with a higher risk for squamous cell carcinoma recurrence, and three-plasma miRNA signature including miR-155-5p, miR-223-3p and miR-126-3p was associated with a higher risk of lung adenocarcinoma progression. However, further studies are needed to fully validate these signatures, so as to investigate the mechanism by which miRNAs enter the bloodstream, to further elucidate the biological significance of miRNAs in the circulation and to evaluate therapeutic response.

## Supporting Information

Figure S1 Heat-map clustering analysis of the deregulated miRNAs expression levels stratified according to NSCLC histology subtypes and either COPD patients or healthy individuals. Average linkage and 1-Pearson correlation as distance metric were used for the clustering. Abbreviations: ADC, adenocarcinoma; COPD, chronic obstructive pulmonary disease; SCC, squamous cell carcinoma.
(TIF)

Figure S2 The expression levels of 13-plasma miRNAs included in our study and detected by qRT-PCR. Paired Student's *t-test* was performed to ascertain statistical significance between the expression levels across groups. Abbreviations: ADC, adenocarcinoma; COPD, chronic obstructive pulmonary disease; SCC, squamous cell carcinoma.
(TIF)

Figure S3 Kaplan-Meier DFS curves for NSCLC patients, independently of histology, stratified according to plasma levels of miR-155-5p (A), miR-20a-5p (B), and miR-152-3p (C). The *P*-values were calculated using the log-rank test between patients with high- and low-fold changes.
(TIF)

Figure S4 Kaplan-Meier DFS curves for NSCLC patients, independently of histology, stratified according to plasma levels of miR-223-3p (A), miR-191-5p (B), miR-320-3p (C), miR-126-3p (D), miR-145-5p (E), miR-199a-5p (F), miR-24-3p (G), miR-25-3p (H), miR-296-5p (I), and let-7f-5p (J). The *P*-values were calculated using the log-rank test between patients with high- and low-fold changes.
(TIF)

Table S1 The panel of miRNAs included in the study.
(DOC)

Table S2 Correlation of plasma miRNAs expression with clinicopathological parameters of NSCLC patients.
(DOC)
