# Supplementary figures and images for: Two Panels of Plasma MicroRNAs as Non-Invasive Biomarkers for Prediction of Recurrence in Resectable NSCLC
Source: PLoS One. 2013 Jan 16;8(1):e54596. doi: 10.1371/journal.pone.0054596 (PMC3546982; doi:10.1371/journal.pone.0054596)

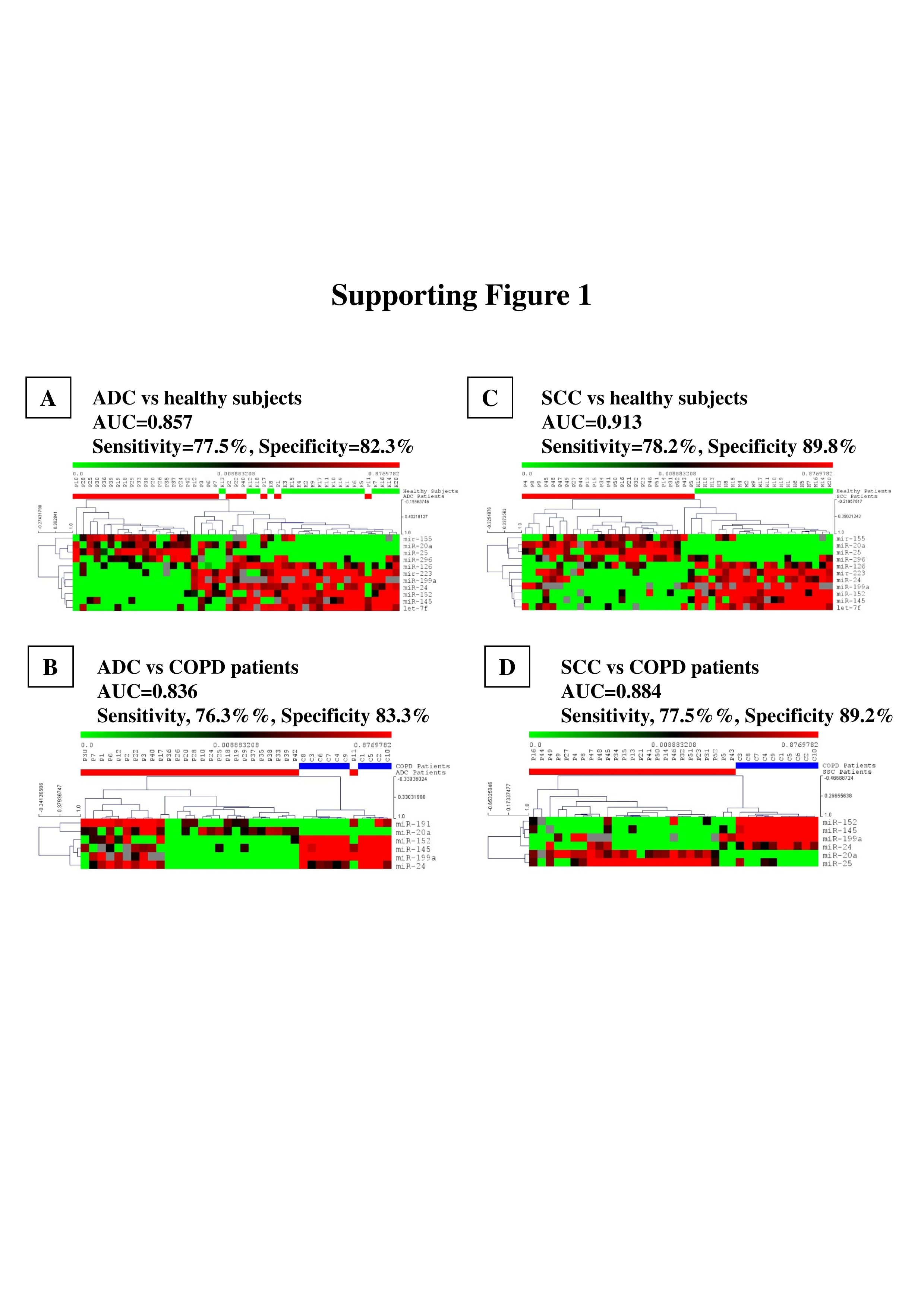

Supplement: Figure S1 — Heat-map clustering analysis of the deregulated miRNAs expression levels stratified according to NSCLC histology subtypes and either COPD patients or healthy individuals. Average linkage and 1-Pearson correlation as distance metric were used for the clustering. Abbreviations: ADC, adenocarcinoma; COPD, chronic obstructive pulmonary disease; SCC, squamous cell carcinoma. (TIF) [file pone.0054596.s001.tif]

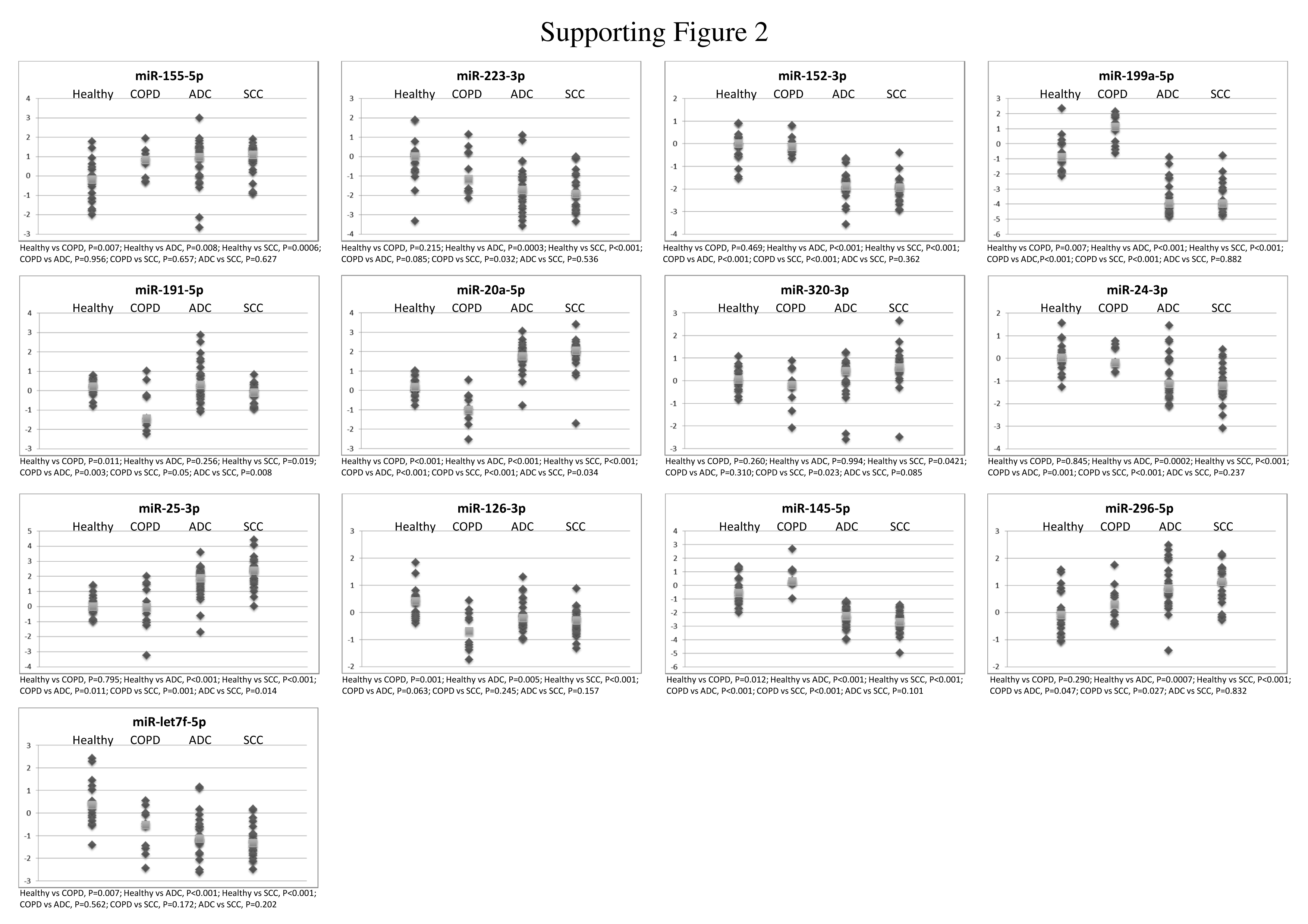

Supplement: Figure S2 — The expression levels of 13-plasma miRNAs included in our study and detected by qRT-PCR. Paired Student's t-test was performed to ascertain statistical significance between the expression levels across groups. Abbreviations: ADC, adenocarcinoma; COPD, chronic obstructive pulmonary disease; SCC, squamous cell carcinoma. (TIF) [file pone.0054596.s002.tif]

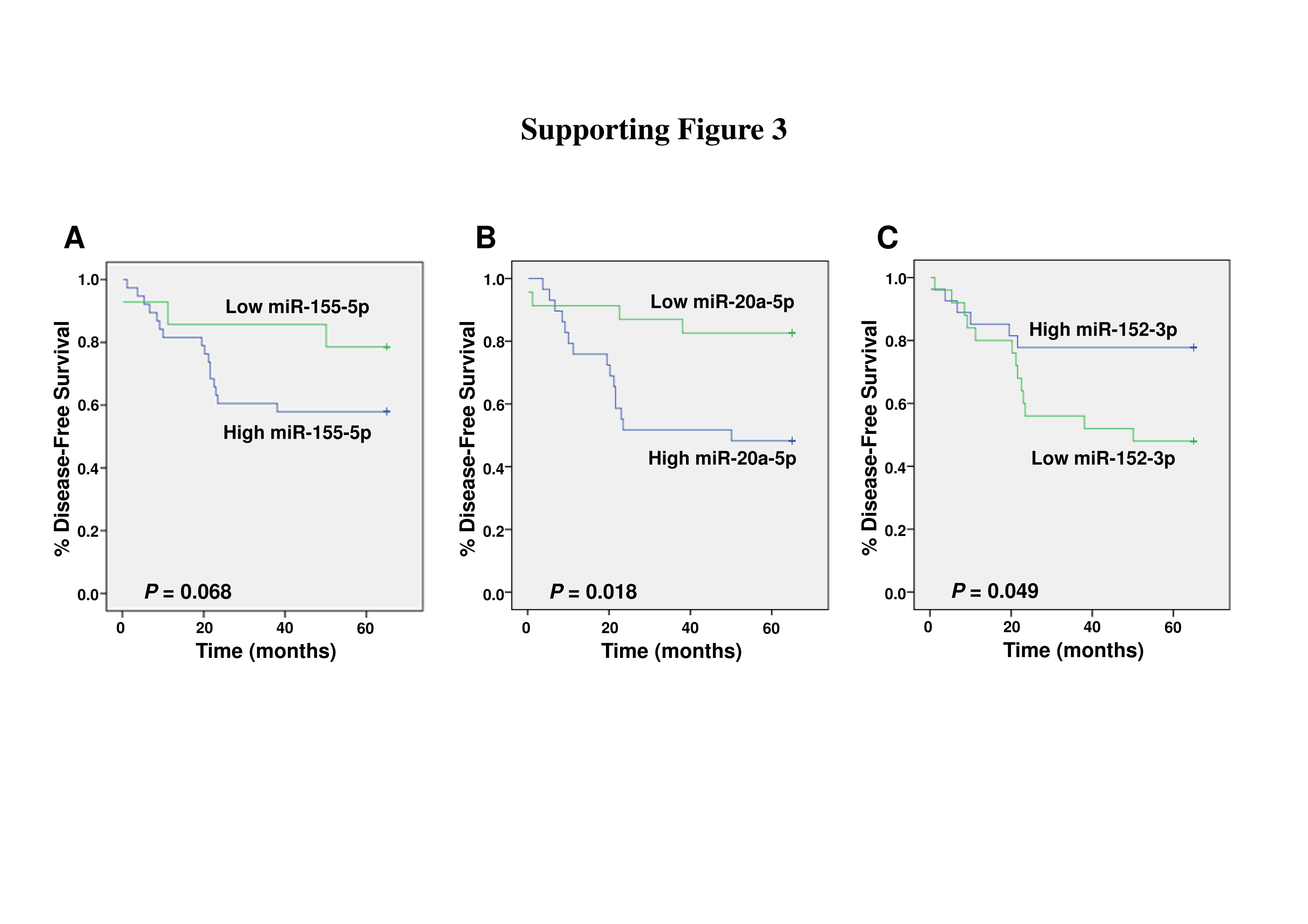

Supplement: Figure S3 — Kaplan-Meier DFS curves for NSCLC patients, independently of histology, stratified according to plasma levels of miR-155-5p (A), miR-20a-5p (B), and miR-152-3p (C). The P-values were calculated using the log-rank test between patients with high- and low-fold changes. (TIF) [file pone.0054596.s003.tif]

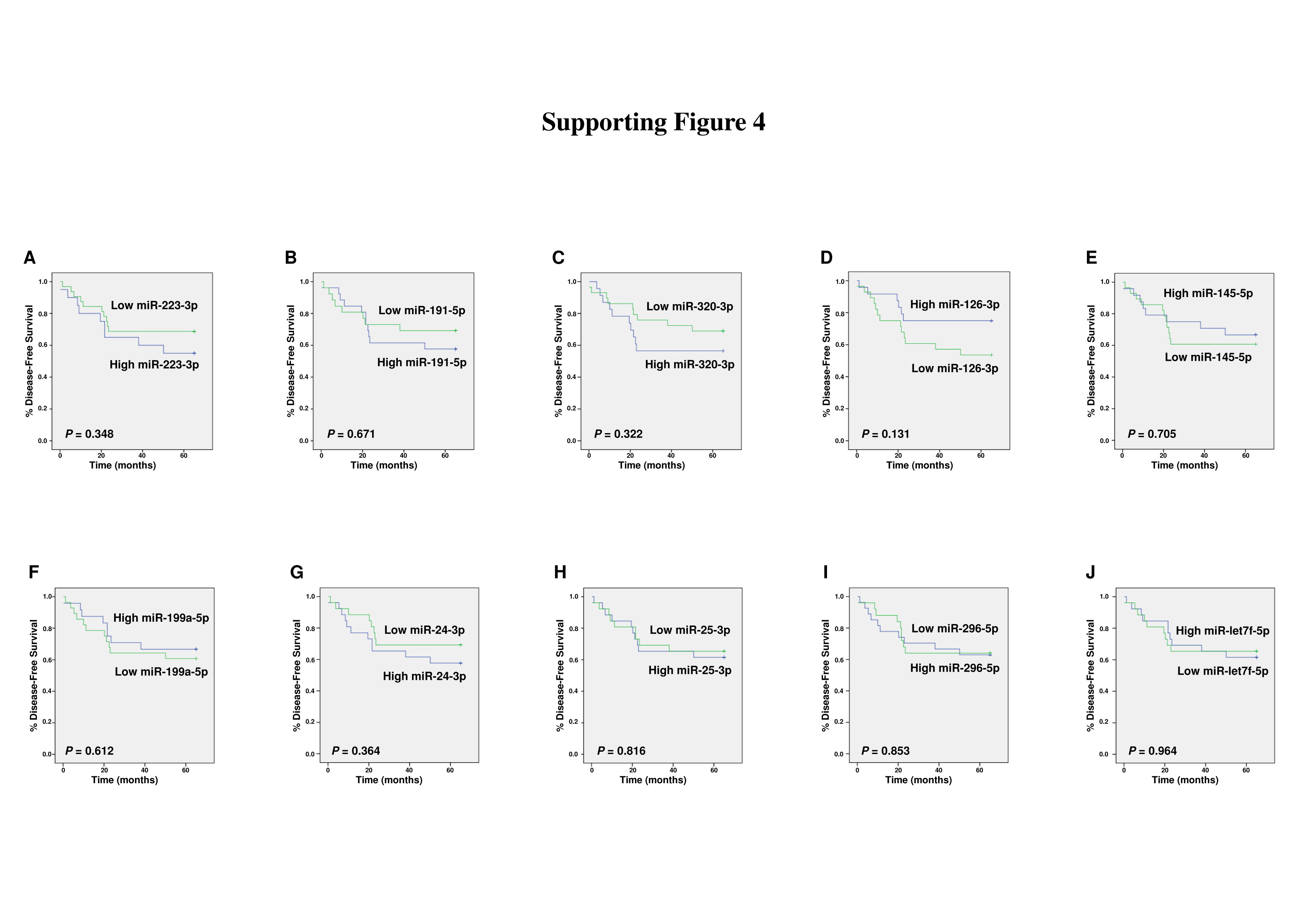

Supplement: Figure S4 — Kaplan-Meier DFS curves for NSCLC patients, independently of histology, stratified according to plasma levels of miR-223-3p (A), miR-191-5p (B), miR-320-3p (C), miR-126-3p (D), miR-145-5p (E), miR-199a-5p (F), miR-24-3p (G), miR-25-3p (H), miR-296-5p (I), and let-7f-5p (J). The P-values were calculated using the log-rank test between patients with high- and low-fold changes. (TIF) [file pone.0054596.s004.tif]
