# Supplementary material for: Two Panels of Plasma MicroRNAs as Non-Invasive Biomarkers for Prediction of Recurrence in Resectable NSCLC
Source: PLoS One. 2013 Jan 16;8(1):e54596. doi: 10.1371/journal.pone.0054596 (PMC3546982; doi:10.1371/journal.pone.0054596)
Supplement: Table S1 — The panel of miRNAs included in the study. (DOC) [file pone.0054596.s005.doc]

| **miRNA** | **Deregulated in NSCLC** | **References** |
| --- | --- | --- |
| **mir-20a-5p** | Up/tissue SCC |  |
| **miR-24-3p** | Down/tissue and serum NSCLC NOS |  |
| **miR-25-3p** | Up/*EGFR* mutated ADC & SCC |  |
| **miR-96-5p** | Up/tissue and plasma SCC |  |
| **let-7f-5p** | Down/tissue ADC |  |
| **miR-126-3p** | Down/tissue and plasma ADC |  |
| **miR-129-5p** | Down/tissue NSCLC NOS |  |
| **miR-145-5p** | Down/tissue and plasma NSCLC NOS |  |
| **miR-152-3p** | Down/tissue and plasma SCC |  |
| **miR-155-5p** | Up/tissue ADC |  |
| **miR-191-5p** | Up/tissue NSCLC NOS/SCC |  |
| **miR-199a-5p** | Down/tissue ADC |  |
| **mir-223-3p** | Up/plasma NSCLC NOS |  |
| **mir-296-5p** | Down/tissue NSCLC NOS |  |
| **mir-320-3p** | Up/serum NSCLC NOS |  |
| **mir-373-5p** | Up/tissue NSCLC NOS |  |
| **mir-516-5p** | Down/tissue NSCLC NOS |  |
| Abbreviations: ADC, adenocarcinoma; NSCLC, non-small cell lung cancer; NOS, not otherwise specified; SCC, squamous cell carcinoma. | | |

**Supporting Table 1**

Supporting Reference List

1. Hayashita Y, Osada H, Tatematsu Y, Yamada H, Yanagisawa K, et al. (2005) A polycistronic microRNA cluster, miR-17-92, is overexpressed in human lung cancers and enhances cell proliferation. Cancer Res 65: 9628-9632.

2. Huang W, Hu J, Yang DW, Fan XT, Jin Y, et al. (2012) Two MicroRNA Panels to Discriminate Three Subtypes of Lung Carcinoma in Bronchial Brushing Specimens. Am J Respir Crit Care Med.

3. Chen X, Ba Y, Ma L, Cai X, Yin Y, et al. (2008) Characterization of microRNAs in serum: a novel class of biomarkers for diagnosis of cancer and other diseases. Cell Res 18: 997-1006.

4. Volinia S, Calin GA, Liu CG, Ambs S, Cimmino A, et al. (2006) A microRNA expression signature of human solid tumors defines cancer gene targets. Proc Natl Acad Sci U S A 103: 2257-2261.

5. Schrauder MG, Strick R, Schulz-Wendtland R, Strissel PL, Kahmann L, et al. (2012) Circulating micro-RNAs as potential blood-based markers for early stage breast cancer detection. PLoS One 7: e29770.

6. Dacic S, Kelly L, Shuai Y, Nikiforova MN (2010) miRNA expression profiling of lung adenocarcinomas: correlation with mutational status. Mod Pathol 23: 1577-1582.

7. Chen X, Hu Z, Wang W, Ba Y, Ma L, et al. (2012) Identification of ten serum microRNAs from a genome-wide serum microRNA expression profile as novel noninvasive biomarkers for nonsmall cell lung cancer diagnosis. Int J Cancer 130: 1620-1628.

8. Zhu W, Liu X, He J, Chen D, Hunag Y, et al. (2011) Overexpression of members of the microRNA-183 family is a risk factor for lung cancer: a case control study. BMC Cancer 11: 393.

9. Takamizawa J, Konishi H, Yanagisawa K, Tomida S, Osada H, et al. (2004) Reduced expression of the let-7 microRNAs in human lung cancers in association with shortened postoperative survival. Cancer Res 64: 3753-3756.

10. Shen J, Todd NW, Zhang H, Yu L, Lingxiao X, et al. (2011) Plasma microRNAs as potential biomarkers for non-small-cell lung cancer. Lab Invest 91: 579-587.

11. Yanaihara N, Caplen N, Bowman E, Seike M, Kumamoto K, et al. (2006) Unique microRNA molecular profiles in lung cancer diagnosis and prognosis. Cancer Cell 9: 189-198.

12. Hennessey PT, Sanford T, Choudhary A, Mydlarz WW, Brown D, et al. (2012) Serum microRNA biomarkers for detection of non-small cell lung cancer. PLoS One 7: e32307.

13. Patnaik SK, Kannisto E, Knudsen S, Yendamuri S (2010) Evaluation of microRNA expression profiles that may predict recurrence of localized stage I non-small cell lung cancer after surgical resection. Cancer Res 70: 36-45.

14. Raponi M, Dossey L, Jatkoe T, Wu X, Chen G, et al. (2009) MicroRNA classifiers for predicting prognosis of squamous cell lung cancer. Cancer Res 69: 5776-5783.

15. Kim S, Lee UJ, Kim MN, Lee EJ, Kim JY, et al. (2008) MicroRNA miR-199a* regulates the MET proto-oncogene and the downstream extracellular signal-regulated kinase 2 (ERK2). J Biol Chem 283: 18158-18166.

16. Silva J, Garcia V, Zaballos A, Provencio M, Lombardia L, et al. (2011) Vesicle-related microRNAs in plasma of nonsmall cell lung cancer patients and correlation with survival. Eur Respir J 37: 617-623.

17. Vaira V, Faversani A, Dohi T, Montorsi M, Augello C, et al. (2012) miR-296 regulation of a cell polarity-cell plasticity module controls tumor progression. Oncogene 31: 27-38.

18. Seike M, Goto A, Okano T, Bowman ED, Schetter AJ, et al. (2009) MiR-21 is an EGFR-regulated anti-apoptotic factor in lung cancer in never-smokers. Proc Natl Acad Sci U S A 106: 12085-12090.
