# Supplementary material for: Two Panels of Plasma MicroRNAs as Non-Invasive Biomarkers for Prediction of Recurrence in Resectable NSCLC
Source: PLoS One. 2013 Jan 16;8(1):e54596. doi: 10.1371/journal.pone.0054596 (PMC3546982; doi:10.1371/journal.pone.0054596)
Supplement: Table S2 — Correlation of plasma miRNAs expression with clinicopathological parameters of NSCLC patients. (DOC) [file pone.0054596.s006.doc]

**Supporting Table 2**

| **Variables** | **miR-155-5p** | | **P** | **miR-223-3p** | | **P** | **miR-191-5p** | | **P** | **miR-320-3p** | | **P** | **miR-126-3p** | | **P** | **miR-145-5p** | | **P** | **miR-152-3p** | | **P** | **miR-199a-5p** | | **P** | **miR-20a-5p** | | **P** | **miR-24-3p** | | **P** | **miR-25-3p** | | **P** | **miR-296-5p** | | **P** | **Let7f-5p** | | **P** |
| --- | --- | --- | --- | --- | --- | --- | --- | --- | --- | --- | --- | --- | --- | --- | --- | --- | --- | --- | --- | --- | --- | --- | --- | --- | --- | --- | --- | --- | --- | --- | --- | --- | --- | --- | --- | --- | --- | --- | --- |
|  | **Low** | **High** |  | **Low** | **High** |  | **Low** | **Low** |  | **Low** | **High** |  | **Low** | **High** |  | **Low** | **High** |  | **Low** | **High** |  | **Low** | **High** |  | **Low** | **High** |  | **Low** | **High** |  | **Low** | **High** |  | **Low** | **High** |  | **Low** | **High** |  |
| **Age (*years*)**  **Mean ± SD** | 64.8 ± 1.7 | 65 ± 2.4 | 0.95 | 62.6 ± 5.1 | 67.1 ± 2 | 0.14 | 65.4 ± 1.9 | 63.6 ± 2.3 | 0.55 | 65 ± 1.6 | 63.6 ± 3 | 0.64 | 64.3 ± 2.2 | 65.9 ± 1.9 | 0.62 | 65.7 ± 2.2 | 64.8 ± 1.7 | 0.76 | 65.8 ± 2 | 64 ± 2.1 | 0.52 | 64.6 ± 2.1 | 65.8 ± 2.3 | 0.72 | 64.3 ± 2.1 | 65.5 ± 2 | 0.68 | 63.8 ± 2.1 | 66 ± 1.9 | 0.46 | 63.1 ± 1.8 | 66.3 ± 2.2 | 0.27 | 66.2 ± 2 | 63.5 ± 2 | 0.34 | 65.4 ± 2.1 | 63.7 ± 2 | 0.59 |
| **Sex** |  |  | 0.57 |  |  | 0.32 |  |  | 0.37 |  |  | 0.93 |  |  | 0.72 |  |  | 0.18 |  |  | 0.56 |  |  | 0.80 |  |  | 0.40 |  |  | 0.63 |  |  | 0.52 |  |  | 0.71 |  |  | 0.49 |
| **Male** | 20 | 18 |  | 19 | 20 |  | 24 | 15 |  | 26 | 13 |  | 23 | 16 |  | 21 | 18 |  | 21 | 18 |  | 27 | 12 |  | 23 | 16 |  | 21 | 18 |  | 17 | 22 |  | 19 | 20 |  | 24 | 15 |  |
| **Female** | 5 | 8 |  | 9 | 4 |  | 6 | 7 |  | 8 | 5 |  | 7 | 6 |  | 9 | 14 |  | 5 | 8 |  | 9 | 4 |  | 5 | 8 |  | 6 | 7 |  | 7 | 6 |  | 8 | 5 |  | 6 | 7 |  |
| **Smoking status** |  |  | 0.19 |  |  | 0.51 |  |  | 0.31 |  |  | 0.12 |  |  | 0.40 |  |  | 0.40 |  |  | 0.90 |  |  | 0.27 |  |  | 0.57 |  |  | 0.37 |  |  | 0.59 |  |  | 0.19 |  |  | 0.76 |
| **Never smoked** | 3 | 5 |  | 3 | 5 |  | 4 | 4 |  | 5 | 3 |  | 6 | 2 |  | 6 | 2 |  | 4 | 4 |  | 5 | 3 |  | 3 | 5 |  | 3 | 5 |  | 3 | 8 |  | 6 | 2 |  | 5 | 3 |  |
| **Current of former smokers** | 24 | 20 |  | 25 | 19 |  | 25 | 19 |  | 29 | 15 |  | 24 | 20 |  | 24 | 20 |  | 22 | 22 |  | 27 | 17 |  | 25 | 19 |  | 24 | 20 |  | 21 | 23 |  | 21 | 23 |  | 25 | 19 |  |
| **Histological type** |  |  | 0.58 |  |  | 0.97 |  |  | 0.023 |  |  | 0.17 |  |  | 0.55 |  |  | 0.25 |  |  | 0.95 |  |  | 0.09 |  |  | 0.012 |  |  | 0.40 |  |  | 0.04 |  |  | 0.17 |  |  | 0.09 |
| **ADC** | 12 | 15 |  | 15 | 12 |  | 11 | 16 |  | 18 | 9 |  | 14 | 13 |  | 13 | 14 |  | 16 | 11 |  | 11 | 16 |  | 19 | 8 |  | 12 | 15 |  | 16 | 11 |  | 17 | 10 |  | 11 | 16 |  |
| **SCC** | 13 | 12 |  | 14 | 11 |  | 18 | 7 |  | 12 | 13 |  | 15 | 10 |  | 16 | 9 |  | 15 | 10 |  | 16 | 9 |  | 9 | 16 |  | 14 | 11 |  | 8 | 17 |  | 11 | 14 |  | 16 | 9 |  |
| **pTNM stage** |  |  | 0.41 |  |  | 0.34 |  |  | 0.17 |  |  | 0.80 |  |  | 0.62 |  |  | 0.64 |  |  | 0.30 |  |  | 0.41 |  |  | 0.26 |  |  | 0.50 |  |  | 0.94 |  |  | 0.94 |  |  | 0.57 |
| **I** | 13 | 16 |  | 14 | 15 |  | 19 | 10 |  | 20 | 9 |  | 17 | 12 |  | 18 | 11 |  | 17 | 12 |  | 19 | 10 |  | 18 | 11 |  | 13 | 16 |  | 14 | 15 |  | 15 | 14 |  | 14 | 15 |  |
| **II** | 9 | 7 |  | 11 | 5 |  | 8 | 8 |  | 14 | 6 |  | 8 | 8 |  | 9 | 7 |  | 7 | 9 |  | 13 | 3 |  | 8 | 8 |  | 10 | 6 |  | 7 | 9 |  | 9 | 7 |  | 10 | 6 |  |
| **III** | 5 | 2 |  | 3 | 4 |  | 2 | 5 |  | 4 | 3 |  | 5 | 2 |  | 3 | 4 |  | 2 | 5 |  | 4 | 3 |  | 2 | 5 |  | 4 | 3 |  | 3 | 4 |  | 3 | 4 |  | 3 | 4 |  |
| **Histological grade** |  |  | 0.37 |  |  | 0.80 |  |  | 0.43 |  |  | 0.69 |  |  | 0.82 |  |  | 0.60 |  |  | 0.93 |  |  | 0.96 |  |  | 0.21 |  |  | 0.37 |  |  | 0.75 |  |  | 0.49 |  |  | 0.30 |
| **Well** | 9 | 13 |  | 12 | 10 |  | 12 | 10 |  | 13 | 9 |  | 13 | 9 |  | 14 | 8 |  | 11 | 11 |  | 15 | 7 |  | 11 | 11 |  | 9 | 13 |  | 11 | 11 |  | 12 | 10 |  | 10 | 12 |  |
| **Moderate** | 11 | 8 |  | 11 | 8 |  | 13 | 6 |  | 13 | 6 |  | 10 | 9 |  | 11 | 8 |  | 10 | 9 |  | 13 | 6 |  | 13 | 6 |  | 11 | 8 |  | 9 | 10 |  | 11 | 8 |  | 13 | 6 |  |
| **Poor** | 7 | 4 |  | 5 | 6 |  | 5 | 6 |  | 8 | 3 |  | 7 | 4 |  | 5 | 6 |  | 5 | 6 |  | 8 | 3 |  | 4 | 7 |  | 7 | 4 |  | 4 | 7 |  | 4 | 7 |  | 7 | 4 |  |
